# Effect and safety of intravenous versus oral acetaminophen after unicompartmental knee replacement: A protocol of randomized controlled study

**DOI:** 10.1097/MD.0000000000021816

**Published:** 2020-08-21

**Authors:** Xiaoming Li, Donghui Guo, Hengjun Wang, Tingting Zhou

**Affiliations:** Department of Orthopedics, The Traditional Chinese Medicine- western Medicine Hospital of Cangzhou, Hebei Province 061001, China.

**Keywords:** intravenous acetaminophen, oral acetaminophen, pain control, randomized controlled trial, study protocol, unicompartmental knee replacement

## Abstract

**Background:**

Due to the soft tissue injury and large amount of bone destruction involved, undesirable postoperative pain remains a challenge for both patients and surgeons after unicompartmental knee replacement (UKR). However, there are no studies comparing the effectiveness of oral and intravenous acetaminophen as part of a standard multimodal perioperative pain regimen after UKR. Thus, this prospective randomized study was conducted to compare pain control outcomes with postoperative oral versus intravenous acetaminophen use in adults undergoing UKR.

**Methods:**

The institutional review board of the Traditional Chinese Medicine- western Medicine Hospital of Cangzhou approved the study protocol. This blinded and randomized study was carried out in accordance with the principles of the Helsinki Declaration. We included patients who were scheduled for UKR with an American Society of Anesthesiologists status of I to III, who were mentally competent, and who were able to give consent for enrolment in the study. Patients were randomly assigned on a 1:1 basis to receive either intravenous acetaminophen or oral acetaminophen. We ensured that the patients, care providers, and outcome assessors were blinded to the group assignment during the study period. Primary outcomes were postoperative pain at rest and during motion (knee flexion of 45°) measured using a visual analog scale score. Secondary outcomes included morphine consumption at 24, 48, and 72 hours after surgery, length of hospital stay, range of motion, daily ambulation distance, and adverse events occurrence. All statistical analyses were performed using SPSS 25.0. Differences associated with a *P* value of <.05 were considered statistically significant.

**Results:**

It was hypothesized that patients receiving intravenous acetaminophen would exhibit similar postoperative outcomes compared with patients receiving oral acetaminophen.

**Trial registration:**

This study was registered in Research Registry (researchregistry5825).

## 1 Introduction

Anteromedial knee osteoarthritis is a distinct clinicopathological entity which often leads to disabling pain and limitation of range of motion.^[1]^ If conservative treatment fails, unicompartmental knee replacement (UKR) is a good treatment option achieving good-to-excellent results and a 10-year survivorship up to 96%.^[2,3]^ The number of UKRs performed has increased over the last decade by 30%, and several studies have demonstrated shorter hospital stays, decreased perioperative morbidity, faster functional recovery, increased range of motion, and improved knee kinematics vs total knee arthroplasty.^[4,5]^ However, due to the soft tissue injury and large amount of bone destruction involved, undesirable postoperative pain remains a challenge for both patients and surgeons after UKR.^[6]^

Acetaminophen, also referred to as paracetamol, is seen as a viable adjunctive medication to help reduce the need for opioids following total joint arthroplasty.^[7,8]^ Although acetaminophen has traditionally been administered orally, an intravenous preparation has recently become available. The intravenous formulation of acetaminophen was approved in the United States in 2010 for management of mild-to-moderate pain, moderate-to-severe pain with adjunctive analgesics and reduction of fever.^[9–11]^ When compared to oral acetaminophen, intravenous administration has earlier and greater bloodbrain barrier penetration and twice the cerebrospinal fluid bioavailability over the first 6 hours. However, oral acetaminophen is cheaper and more convenient than intravenous formulation.^[10]^

Some of the high quality studies have confirmed that oral and intravenous preparation, in total knee and hip arthroplasties, can achieve similar postoperative analgesic effect.^[10–14]^ However, there are no studies comparing the effectiveness of oral and intravenous acetaminophen as part of a standard multimodal perioperative pain regimen after UKR. Therefore, the question of potential analgesic and motor-sparing benefits of oral versus intravenous acetaminophen in the setting of UKR remains unanswered. Thus, this prospective randomized study was conducted to compare pain control outcomes with postoperative oral versus intravenous acetaminophen use in adults undergoing UKR. It was hypothesized that patients receiving intravenous acetaminophen would exhibit similar postoperative outcomes compared with patients receiving oral acetaminophen.

## 2 Material and method

**Figure 1:**
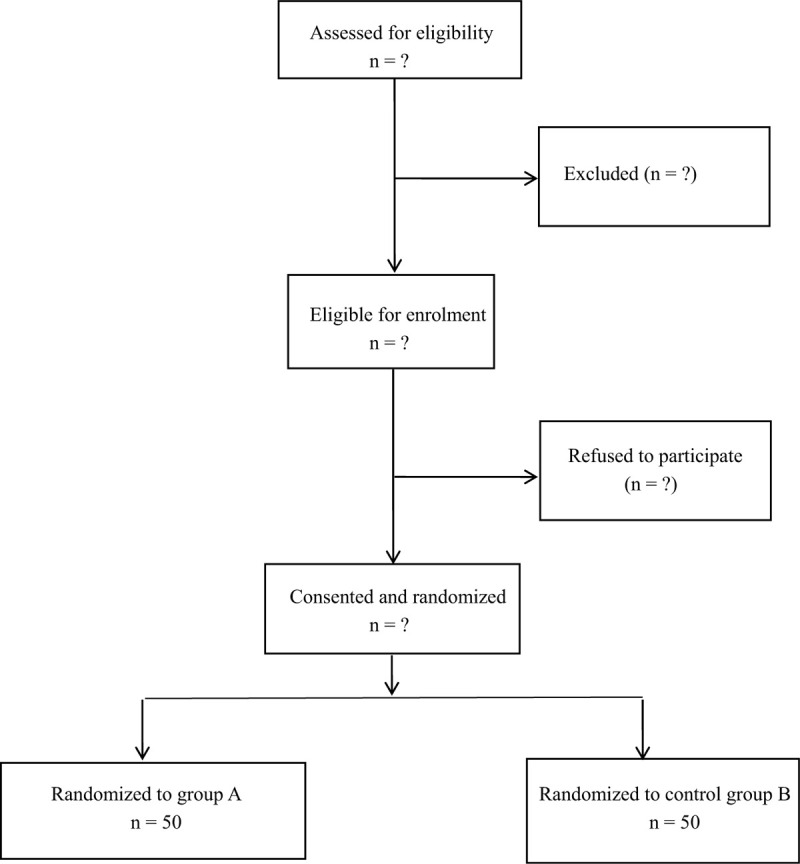
Consolidated Standards of Reporting Trials (CONSORT) diagram of patient flow through the study.

The institutional review board of the Traditional Chinese Medicine- western Medicine Hospital of Cangzhou approved the study protocol (sop/013/01.0). This blinded and randomised study was carried out in accordance with the principles of the Helsinki Declaration. This trial was also registered in Research Registry (researchregistry5825). The flowchart of this trial is shown in Figure 1.

### 2.1 Participants

We included patients who were scheduled for UKR with an American Society of Anesthesiologists status of I to III, who were mentally competent, and who were able to give consent for enrolment in the study. Patients were excluded if they had significant patellofemoral or lateral osteoarthritis, secondary arthritis due to rheumatoid arthritis or trauma, or osteonecrosis. We also excluded patients with a body mass index of 40 kg/m^2^ or more, those undergoing revision knee arthroplasty, those with impaired kidney function or a coagulopathy, and those with chronic pain syndromes or chronic opioid use.

### 2.2 Randomization and blinding

Patients were randomly assigned on a 1:1 basis to receive either intravenous acetaminophen or oral acetaminophen. Randomized numbers ranging from 0 to 99 were generated using computer software. On enrollment, a sealed envelope was selected in the pharmacy department by allocating staff who did not take part in surgery or assessment of outcome. Patients with even numbers were allocated to the group scheduled to receive intravenous acetaminophen, and those with odd numbers were allocated to receive oral acetaminophen. We ensured that the patients, care providers, and outcome assessors were blinded to the group assignment during the study period. They were all unaware of the randomization given by the allocating staff.

### 2.3 Intervention and control

In the intravenous acetaminophen group, 1000 mg of intravenous acetaminophen was administered at 22:00 on the day of surgery, at 04:00, 10:00, 16:00, and 22:00 two days after surgery, and at 04:00 three days after surgery. An intravenous dose of 15 mg/kg acetaminophen was used for patients with body weight <50 kg. In the oral acetaminophen group, capsules of acetaminophen were administered in the same dose and manner as described for intravenous acetaminophen.

### 2.4 Surgical technique

The implant sizes were selected based on pre-operative templating. A paramedial quadriceps sparing incision and approach were utilized and UKR performed using the conventional instrumentation, in accordance with the operative technique. A vertical tibial cut was performed using a hand-held reciprocating saw, with reference to the tibial cutting guide and appropriate anatomical landmarks. The horizontal tibial cut was performed using an oscillating saw. An intramedullary reference was used to position the femoral cutting guide and the posterior femoral cut made using an oscillating saw. The distal femur was milled and the flexion/extension gaps balanced. The bone surfaces were prepared and the cobalt chrome implants cemented and a polyethylene mobile bearing inserted.

### 2.5 Outcome measures

Primary outcomes were postoperative pain at rest and during motion (knee flexion of 45°) measured using a visual analog scale (VAS) score (on a scale of 0–10, where 0 indicates no pain and 10 indicates severe pain). Pain at rest was measured at 12, 24, 48, and 72 hours after surgery, and pain during motion was measured at 12, 24, 48, and 72 hours after surgery. The supplemental use of morphine to treat postoperative pain was also recorded. Secondary outcomes included morphine consumption at 24, 48, and 72 hours after surgery, length of hospital stay, range of motion, daily ambulation distance, and adverse events occurrence.

### 2.6 Statistical analysis

All data are presented as means and standard deviations, unless otherwise indicated. As patient demographic characteristics were normally distributed, continuous data (e.g., age and body mass index) were analyzed using Student's *t* test, while categorical data (e.g., gender and target side of the body) were analyzed using the chi-squared test. Clinical outcomes measured after surgery was not normally distributed and so were analyzed using the Mann-Whitney U-test for continuous data (e.g., pain score, morphine consumption, knee range of motion) and the chi-squared test for categorical data (e.g., adverse events occurrence). All statistical analyses were performed using SPSS 25.0. Differences associated with a *P* value of <.05 were considered statistically significant.

### 2.7 Sample size calculation

We estimate that with 50 participants in each group, the study will have more than 80% power to detect a clinically important difference between the groups in regard to the change in the pain score evaluated with the VAS. This is assuming a mean intergroup difference in score of 20 mm based on previous literature and a pooled standard deviation of 35 mm on the basis of preliminary data at an alpha level of 5%. Based on this estimation, a total of 120 patients are needed with an allowance for 10% drop-out.

## 3 Discussion

This randomized, double-blinded trial compared intravenous to oral acetaminophen after UKR among patients receiving a comprehensive opioid-sparing multimodal analgesia regimen. It was hypothesized that patients receiving intravenous acetaminophen would exhibit similar postoperative outcomes compared with patients receiving oral acetaminophen. The strengths of this blinded, randomized, controlled trial include the use of comprehensive multimodal analgesia, consisting of neuraxial anesthesia; postoperative, opioid-free, patient-controlled analgesia; and nonsteroidal anti-inflammatory drug administration. Opioids were provided only as needed. The biggest design flaws was the absence of a placebo control, which implies that the natural course of postoperative pain or a general placebo effect cannot be ruled out to explain the observed effects.

## Author contributions

Xiaoming Li and Donghui Guo wrote the first draft of the article of the study protocol. Hengjun Wang and Tingting Zhou are responsible for review and editing. Xiaoming Li, Hengjun Wang, Donghui Guo and Tingting Zhou are responsible for managing the project and conducting a formal analysis. Donghui Guo, Hengjun Wang and Tingting Zhou are responsible for data curation. Xiaoming Li received the funding to ensure that this study could go forward. All authors have contributed to the design and implementation of the study.
